# LMNA E82K Mutation Activates FAS and Mitochondrial Pathways of Apoptosis in Heart Tissue Specific Transgenic Mice

**DOI:** 10.1371/journal.pone.0015167

**Published:** 2010-12-06

**Authors:** Dan Lu, Hong Lian, Xiaojuan Zhang, Haitao Shao, Lan Huang, Chuan Qin, Lianfeng Zhang

**Affiliations:** 1 Key Laboratory of Human Disease Comparative Medicine, Ministry of Health, Institute of Laboratory Animal Science, Chinese Academy of Medical Sciences & Comparative Medical Center, Peking Union Medical College, Beijing, China; 2 Key Laboratory of Human Disease Animal Model, State Administration of Traditional Chinese Medicine, Institute of Laboratory Animal Science, Chinese Academy of Medical Sciences & Comparative Medical Center, Peking Union Medical College, Beijing, China; Wayne State University School of Medicine, United States of America

## Abstract

The lamin A/C (LMNA), nuclear intermediate filament proteins, is a basic component of the nuclear lamina. Mutations in LMNA are associated with a broad range of laminopathies, congenital diseases affecting tissue regeneration and homeostasis. Heart tissue specific transgenic mice of human LMNA E82K, a mutation causing dilated cardiomyopathy, were generated. *Lmna* ^E82K^ transgenic mouse lines exhibited thin-walled, dilated left and right ventricles, a progressive decrease of contractile function assessed by echocardiography. Abnormalities of the conduction system, myocytes disarray, collagen accumulation and increased levels of B-type natriuretic peptide (BNP), procollagen type III α1 (Col3α1) and skeletal muscle actin α1 (Actα1) were detected in the hearts of *Lmna* ^E82K^ transgenic mice. The LMNA E82K mutation caused mislocation of LMNA in the nucleus and swollen mitochondria with loss of critae, together with the loss of nuclear envelope integrity. Most interestingly, we found that the level of apoptosis was 8.5-fold higher in the *Lmna* ^E82K^ transgenic mice than that of non-transgenic (NTG) mice. In the presence of the LMNA E82K, both of FAS and mitochondrial pathways of apoptosis were activated consistent with the increase of FAS expression, the release of cytochrome *c* from mitochondria to cytosol and activation of caspase-8, -9 and -3. Our results suggested that the apoptosis, at least for the LMNA E82K or the mutations in the rod region of Lamin A/C, might be an important mechanism causing continuous loss of myocytes and lead to myocardial dysfunction. It could be a potential therapeutic means to suppress and/or prevent inappropriate cardiac cell death in patients carrying LMNA mutation.

## Introduction

The LMNA gene is alternatively spliced to produce the two intermediate filament proteins termed nuclear lamin A/C, which locate to the nuclear lamina, a fibrous structure underlying the inner nuclear membrane [1]. Lamin A/C, emerin and complex which links the nucleoskeleton and cytoskeleton (LINC) form a variety of macro-protein complexes at the nuclear envelope and together cross-link the nuclear skeleton to the cytoskeleton. These protein complexes function to maintain nuclear architecture and stability and cellular tensegrity [2]–[4]. The lamins play important roles in DNA replication, chromatin organization, regulation of gene expression, spatial organization of the nuclear pore and the correct anchorage of the nuclear envelope proteins, cell development, differentiation and apoptosis [5].

The mutations in the LMNA gene has been shown to cause at least nine different autosomal recessive and dominant genetic diseases, collectively called laminopathies [6], [7]. More than 40 mutations in the LMNA gene have been shown to be involved in the severity of the cardiac symptoms, characterized by conduction defect, arrhythmias, left ventricular (LV) dysfunction, dilation with heart failure or sudden death [8]–[13].

Lamin A/C plays a crucial role in many cellular activities, but it is poorly understood why and how different mutants cause such diverse phenotypes in specific tissues, but other tissues are apparently unaffected [14], and the identification of the precise molecular mechanisms of LMNA mutations leading to laminopathies is also critical for developing new therapeutic strategies to prevent cardiac dysfunction and sudden death.

A novel mutation E82K in lamin A/C gene has been found to cause dilated cardiomyopathy (DCM) in a large Chinese pedigree with 50 family members [15]. In the current paper, a heart tissue specific transgenic mice expressing LMNA E82K was generated and the mechanism causing dilated cardiomyopathy for this mutation were investigated in the transgenic mice.

## Results

### Generation of the transgenic mice

**Figure 1:**
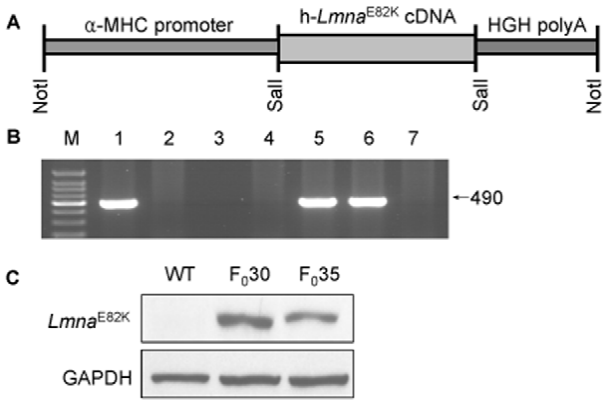
Generation of the transgenic mice. (A) The LMNA E82K transgenic construct was generated by inserting the target genes under the control of the α-MHC heart tissue specific promoter and the transgenic mice were created following microinjection. (B) Screening of mouse genomic DNA by PCR for the presence of LMNA E82K gene. M: molecular weight marker. Lane 1: positive control of *Lmna* ^E82K^ transgenic mice; lane 2: negative control; lane 3: blank control; lane 5 and 6: positive *Lmna* ^E82K^ transgenic mice; lane 4 and 7: negative transgenic mice. (C) The mouse lines, founder 30 and 35, with over-expression of LMNA E82K were selected by the western blot procedure using GAPDH as normalization.

C57BL/6J mice carrying the human LMNA E82K gene were established (Fig. 1). Two lines of *Lmna* ^E82K^ transgenic mice with high level of expression were selected among 53 founders by western blot analysis (Fig. 1C). The *Lmna* ^E82K^ transgenic mice were indistinguishable from their non-transgenic (NTG) littermates at birth and young-age. The death of the two transgenic lines occurred from 3 months old and mortality was 15.8% (3 of 19 for founder 30) and 11.1% (2 of 18 for founder 35) at 10 months of age respectively, while no death was observed in NTG mice.

### *Lmna* ^E82K^ caused dysfunction of heart in transgenic mice

**Figure 2:**
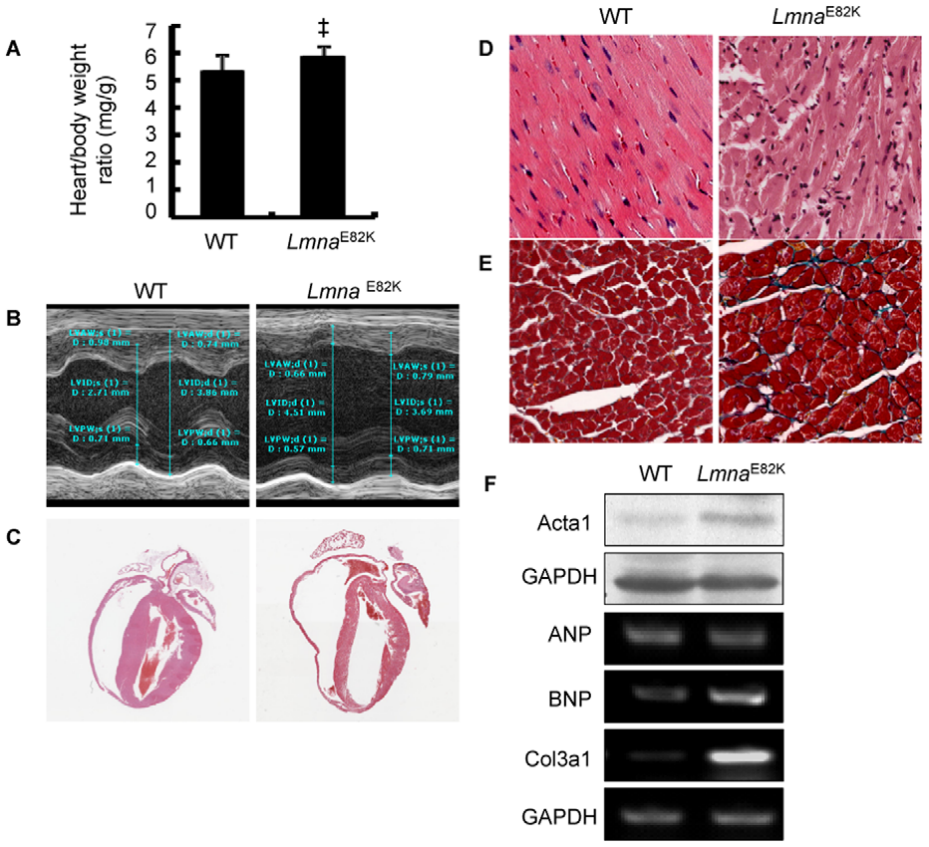
The effects of LMNA E82K on heart dimensions and hypertrophic marker expression in the transgenic mice. (A) Heart weight to body weight ratio was determined (*n* = 14, ^‡^ *P*<0.01 *versus* NTG mice). (B) Representative M-mode echocardiographic images of the LV long-axis of the NTG and *Lmna* ^E82K^ transgenic mice. (C) H&E staining patterns of the whole-heart longitudinal sections from 6 months old NTG and *Lmna* ^E82K^ transgenic mice (magnification ×20). (D) Magnification of H&E stained sections of LV (magnification ×400). (E) Magnification of Masson trichrome stained sections of LV in NTG and *Lmna* ^E82K^ transgenic mice (magnification ×400). (F) Expression of Actα1, ANP, BNP and Col3α1 were detected by western blot and RT-PCR procedure using GADPH as normalization.

Ventricular size and function of the two transgenic lines were assessed using echocardiography. The parameters of M-mode echocardiography from the NTG and *Lmna* ^E82K^ transgenic mice at 2, 4, 6 and 8 months of age were summarized in Table 1 and Table S1. LMNA E82K mutation significantly increased the heart to body weight ratio by 10% (Fig. 2A, *n* = 14, *P*<0.01) by gross morphology examination. The representative M-mode echocardiograms from founder 35 at 6 months of age were shown in figure 2B. To sum up, the LMNA E82K hearts exhibited thin-walled and dilated left and right ventricles when compared with NTG hearts (Table 1). *Lmna* ^E82K^ transgenic mice developed a progressive LV dilation and dysfunction associated with a progressive decrease of contractile function, evidenced by decreased LV percent fractional shortening (FS %) which exhibited a significance from 2 months of age compared with NTG mice (Table 1 and Table S1, *P*<0.01).

**Table 1:** Echocardiographic characteristics of NTG and *Lmna* ^E82K^ transgenic mice at 6 months of age.

Parameters	NTG	*Lmna* ^E82K^ (line 30)	*Lmna* ^E82K^ (line 35)
**Number of mice**	19	18	18
**LVEDD, mm**	3.92±0.24	4.59±0.29#	4.41±0.23#
**LVESD, mm**	2.71±0.28	3.57±0.47#	3.49±0.26#
**LVEDV, µL**	67.08±9.60	97.34±14.68#	88.74±11.30#
**LVESV, µL**	27.97±7.02	55.05±17.28#	51.03±9.26#
**LVPWD, mm**	0.66±0.09	0.56±0.10‡	0.56±0.06‡
**LVPWS, mm**	0.91±0.09	0.72±0.12#	0.71±0.07#
**LVAWD, mm**	0.74±0.07	0.68±0.08	0.61±0.06#
**LVAWS, mm**	0.94±0.12	0.83±0.13*	0.77±0.09#
**EF%**	58.76±6.38	48.65±8.96#	45.99±9.63#
**FS%**	30.86±4.46	24.76±5.38#	23.28±5.08#
**HR, bpm**	441.72±60.38	423.60±55.85	437.97±60.14

LV: left ventricular; LVEDD: LV end-diastole diameter; LVESD: LV end-systole diameter; LVEDV: LV end-diastolic volume; LVESV: LV end-systole volume; LVPWD: LV posterior wall at end-diastole; LVPWS: LV posterior wall at end-systole; LVAWD: LV anterior wall at end-diastole; LVAWS: LV anterior wall at end-systole; EF%: percent ejection fraction; FS%: percent fractional shortening; HR: heart rate.

*P<0.05 *versus* NTG mice;

‡ *P*<0.01 *versus* NTG mice;

# *P*<0.001 *versus* NTG mice.

Electrocardiography (ECG) measurements were performed in mutant and NTG mice at 7 months of age (Table 2). Compared with WT mice, the PR interval and QRS complex duration had a tendency of increase in both of the transgenic lines, but only the mice generated from founder 35 showed a significant increase in the QRS complex duration (*P*<0.05) in 7 mice with ECG recording. Under light microscopy, myocyte disarray, interstitial fibrosis were observed in the *Lmna* ^E82K^ transgenic mice compared with the NTG mice (Fig. 2C–E). The expression level of hypertrophic markers, BNP, Actα1 and Col3α1, were obviously increased in both of the two transgenic lines compared with the NTG mice (Fig. 2F, the data from founder 35).

**Table 2:** ECG date for NTG and *Lmna* ^82K^ transgenic mice at 7 months of age.

Parameters	NTG	*Lmna* ^E82K^ (line 30)	*Lmna* ^E82K^ (line 35)
**Number of mice**	6	7	7
**PR interval, ms**	0.0269±0.0019	0.0298±0.0028	0.0290±0.0036
**QRS duration, ms**	0.0112±0.0014	0.0129±0.0028	0.0134±0.0017*

*P<0.05 *versus* NTG mice.

### Morphological changes of myocytes and its nucleus in the *Lmna* ^E82K^ transgenic mice

**Figure 3:**
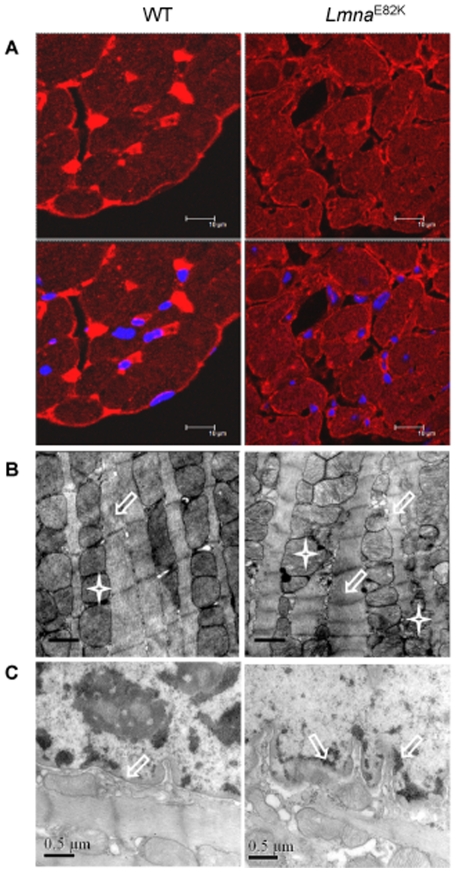
Morphological observation. (A) Immunodetection of LMNA E82K in heart from NTG and *Lmna* ^E82K^ transgenic mice at 6 months of age. Lamin A/C staining appeared red showing the localization of LMNA protein and the sections were counterstained blue with DAPI to visualize the nuclei. Scale bar  = 10 µm. (B) TEM showed abnormal sarcomeres (white hollow arrow) and mitochondria (white star) from LV free walls in the transgenic mice. Scale bars  = 0.5 µm. (C) The collapse and partial fragmentation in nuclear membrane of myocytes in the *Lmna* ^E82K^ transgenic mice were showed.

The immunofluorescence staining of LMNA protein indicated that LMNA E82K was mislocated in the transgenic heart instead of nuclear rim localization in the NTG heart at 7 months of age (Fig. 3A). Ultrastructural observation indicated that enlarged mitochondria and sarcoplasmic reticulum, and loss of nuclear envelope integrity due to the expression of LMNA E82K existed in the *Lmna* ^E82K^ mice compared with that of NTG mice (Fig. 3B and C).

### The FAS and mitochondrial pathways of apoptosis were activated in *Lmna* ^E82K^ transgenic mice

**Figure 4:**
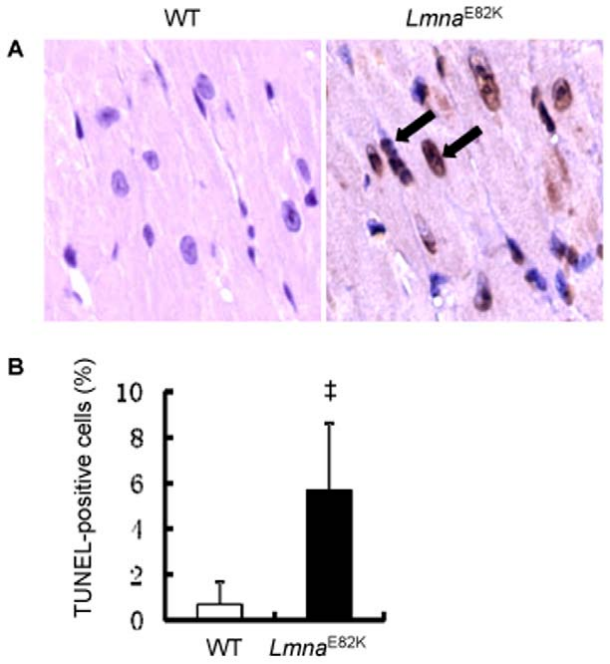
TUNEL assay. (A) Photomicrographs of heart tissue used for TUNEL assay, arrows indicate TUNEL-positive cells (magnification ×800). (B) The quantitative analysis of apoptotic cells in the heart of mice (*n* = 3, ^‡^ *P*<0.01 *versus* NTG mice).

**Figure 5:**
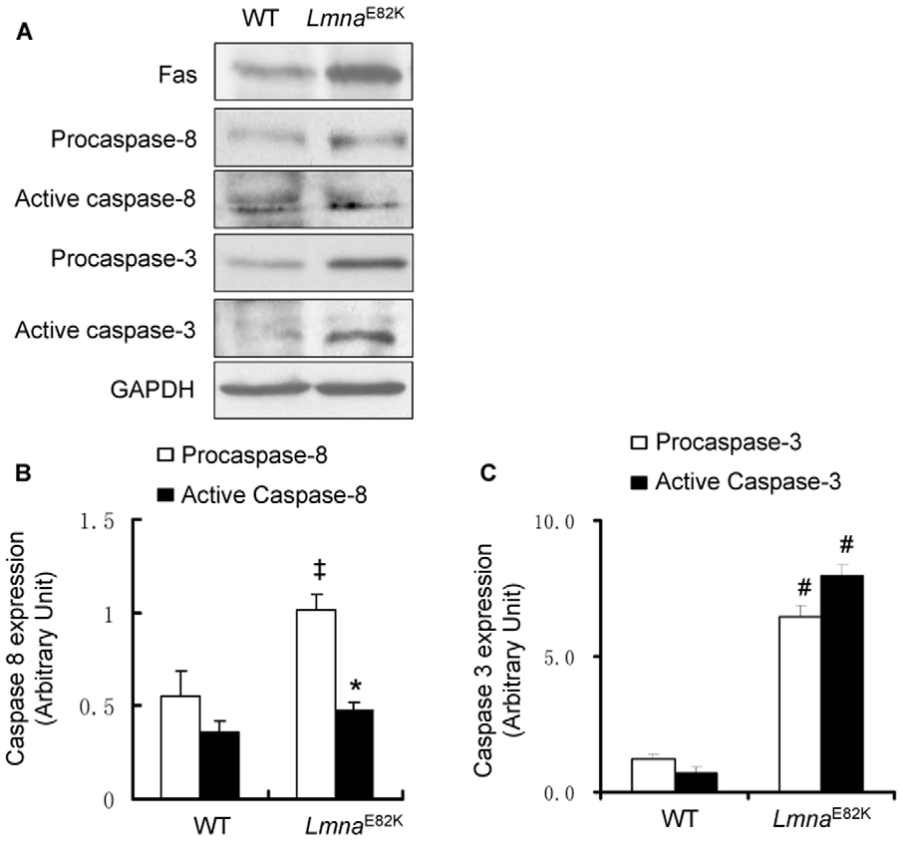
Determination the expression of FAS and caspases. (A) The expression of FAS, caspase-8 and caspase-3 were measured by western blot from the hearts of NTG and *Lmna* ^E82K^ transgenic mice, and a representative experiment was shown. (B–C) The quantitative analysis of caspases using GADPH as normalization (*n* = 3, *P<0.05 *versus* NTG mice; ^‡^ *P*<0.01 *versus* NTG mice;^ #^ *P*<0.001 *versus* NTG mice).

**Figure 6:**
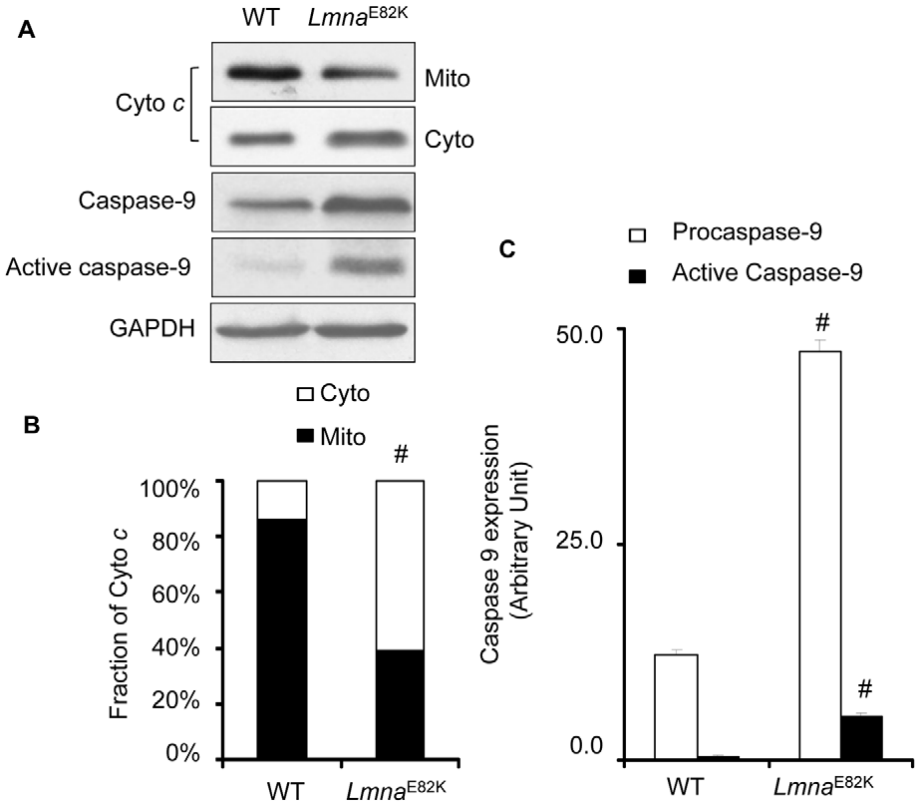
Detection of cytochrome *c* release and activation of caspase-9. (A) Mitochondrial cytochrome *c* release and the expression of caspase-9 were measured by western blot from the hearts of NTG and *Lmna* ^E82K^ transgenic mice, and a representative experiment was shown. (B–C) The quantitative analysis of cytochrome *c* and caspase-9 using GADPH as normalization (*n* = 3, ^#^ *P*<0.001 *versus* NTG mice).

Apoptosis of myocytes was detected in *In situ* terminal dUTP nick end-labeling (TUNEL) assay in heart tissue from *Lmna* ^E82K^ transgenic mice and NTG mice (Fig. 4A). The apoptotic index was increased to 5.67±2.94% in the transgenic mice while it was 0.67±1.03% in the NTG mice (Fig. 4B, *n* = 3, *P*<0.01). We found that the expression of FAS was upregulated significantly in the *Lmna* ^E82K^ transgenic mice (Fig. 5A, *n* = 3, *P*<0.05). The expression of procaspase-8 and the activated caspase-8 were increased 84.8% (Fig. 5A and B, *n* = 3, *P*<0.01) and 32.4% (Fig. 5A and B, *n* = 3, *P*<0.05) respectively. Meanwhile, the expression of procaspase-3 and the activated caspase-3 were 4.4-fold and 10.4-fold higher in the *Lmna* ^E82K^ transgenic mice than that of NTG mice (Fig. 5A and C, *n* = 3, *P*<0.001) respectively. The expression of LMNA E82K also caused the release of cytochrome *c* from mitochondria to cytosol, and results showed that 47% of cytochrome *c* in cytosolic concentrations were accompanied by decreased mitochondrial concentrations in the *Lmna* ^E82K^ transgenic mice (Fig. 6A and B, *n* = 3, *P*<0.001). Meanwhile, the expression of procaspase-9 and the activated caspase-9 were 2.9-fold and 13.5-fold higher in the *Lmna* ^E82K^ transgenic mice than that of NTG mice (Fig. 6A and C, *n* = 3, *P*<0.001) respectively. The results suggested that LMNA E82K mutation induced apoptosis in the heart is likely mediated by both of the FAS and mitochondrial pathways.

## Discussion

Mutations in the LMNA gene are the most common cause of familial dilated cardiomyopathy (FDC) showing to be the severity of the cardiac symptoms, characterized by conduction defect, arrhythmias, LV dysfunction, and dilation with heart failure or sudden death [8]–[13]. A few mice models has been created for lamin A/C knock out or mutations. The LMNA G608G transgenic mice targeted the expression of the Hutchinson-Gilford progeria syndrome (HGPS) mutation in keratin-5-expressing tissue led to a typical phenotype of HGPS [16]. LMNA H222P mutated gene knockin mice exhibited conduction defects, chamber dilation, increased fibrosis and lack of hypertrophy, and also showed muscular dystrophy and death at 4–9 months of age [17]. The patients with heterozygous for the LMNA E82K mutation showed clinical phenotypes of heart dilation and associated with conduction system disease at their onset age of 32 or 33 years [15]. The two *Lmna* ^E82K^ transgenic mice lines exhibited chamber dilation, increased heart weights, increased fibrosis, upregulation of hypertrophic maker expression, nuclear structure defects and conduction defects (Figures 1, 2, 3, Tables 1, 2), which was similar with the phenotypes of the patients carrying the LMNA E82K mutation.

The importance of BNP as a diagnostic and therapeutic modality in cardiovascular disease is well known, it also acts as a local regulator of ventricular remodeling and a modifier of cardiac gene expression [18]–[20]. Actα1 is present in the developing heart and it constitutes up to 20% of the striated actin of the adult heart. Since Actα1 is a multifunctional protein that interacts with many proteins involved in folding, polymerisation, contractility and regulation of contractility, abnormal levels may affect any of those functions [21]. In the normal adult heart, approximately 2 to 4% of the myocardium is made up of collagen. The Col3α1 is one of the essential components of the cardiovascular extracellular matrix, maintaining structural and functional integrity of myocardium and thought to be responsible for abnormal myocardial stiffness and for the impaired pumping capacity of the heart [22]–[23]. The expression of BNP, Actα1 and Col3α1 was unregulated in the two *Lmna* ^E82K^ transgenic mice lines (Fig. 2F).

The death of the transgenic mice occurred from 3 months of age and the mortality of the *Lmna* ^E82K^ transgenic mice was about 15.8% at 10 months of age, while it showed that some patients carrying this mutation died at the age of 42 and 48 years [15], therefore the LMNA E82K mutation caused slight mortality in transgenic mice compared with other mutations of LMNA as LMNA H222P, LMNA M371K and LMNA N195K [17], [24], [25]. LMNA E82K mutation located in the coil 1B domain of central a-helical rod domain of the lamin A and the lamin C proteins, those were conserved regions of the rod domain which have been shown to play crucial roles in the assembly of intermediate filament (IF) dimers into higher order oligomers [26]. Mutations affect this region of IF proteins and may disrupt the interaction between the monomers and are linked to several diseases [27]. We observed that the assembly of the Lamin A/C was disrupted (Fig. 3A), and the integrity of the nuclear envelope was damaged (Fig. 3C) in the *Lmna* ^E82K^ transgenic mice. Members of the intermediate filament superfamily are critical mechanical integrators of the nuclear membrane and the cytoskeleton, protecting the cell from repeated mechanical stress. Mutations in the lamin A/C gene may cause cardiomyopathy by weakening nuclei, which increase the fragility of nuclei and could be particularly harmful to muscle cells. Forces generated during muscle contraction might potentially lead to preferential breakage of nuclei containing a defective nuclear lamina [14].

The accumulation of damaged nuclei as a result of a reduction in load-bearing properties of the nuclear lamina might be a possible mechanism of DCM [28], [29]. The alternate possibility of mechanism for the pathogenesis was the structural weakness of the lamina, which might be a predisposing factor to induce nuclear damage and apoptosis [28], [29]. In the lamin A/C knockout mice, the myocyte apoptosis was observed by 2-fold higher than that of in NTG animals [30], but we found that the level of apoptosis was 8.5-fold higher in the *Lmna* ^E82K^ transgenic mice than that of the NTG mice (Fig. 4A and B). We concluded that LMNA E82K mutation in mice, and probably in humans, disrupted integrity and triggered apoptosis and finally resulted in DCM and heart failure. It was possible that specialized properties of conduction system myocytes made these more susceptible than surrounding myocytes to pro-apoptotic signals triggered by mutated LMNA, and the transgenic mice may developed the conduction defects [31]. Our most interesting finding was that the expression of LMNA E82K in heart tissues increased the expression of FAS, accompanied with the activation of caspase-8 and caspase-3 in *Lmna* ^E82K^ transgenic mice (Fig. 5A–C). The release of cytochrome *c* from mitochondria to cytosol was also induced by the expression of LMNA E82K, followed the activation of caspase-9 (Fig. 6A–C).

FAS, as a member of the death receptor superfamily, plays a central role in the death receptor pathway [32]. After FAS ligand binding, FAS receptors undergo trimerization and recruit FAS-associated death domain (FADD). FAS/FADD complex binds to the initiator caspase-8. According to the cell type, activated caspase-8 may propagate the apoptotic signal either through a direct activation of executioner downstream caspases or via the release of cytochrome *c* from mitochondria [33]–[36]. The involvement of mitochondria in apoptotic processes has already been clearly demonstrated [37], [38], that the release of cytochrome *c* triggers the assembly of Apoptotic protease-activating factor (Apaf-1) and procaspase-9 to form an apoptosome, and procaspase-9 is then autolyticaly cleaved to active caspase-9, which then activates procaspase-3 to active caspase resulting in cleavage of its substrates and apoptosis [39], [40].

Loss of myocytes is a feature of the cardiomyopathic process that contributes to progressive decline in LV function and congestive heart failure [41], [42]. Although a number of stimuli appear to trigger the process of apoptosis in cardiomyocyte. Our results indicated that the two major signaling pathways of apoptosis: the death receptor pathway and the mitochondrial pathway were activated by the expression of LMNA E82K in heart tissue.

It has been indicated that lamin A/C regulates Wnt/β-catenin and MAPK signal pathway, and it also regulates a certain numbers of growth factors and transcription factors, like TGF-β and c-Fos, which regulates differentiation, proliferation and apoptosis in many cell types [43]. The LMNA mutations have been shown to be the severity of the cardiac symptoms, which may cause in diverse mechanisms. The apoptosis, at least for the LMNA E82K or the mutations in the rod region of Lamin A/C, might be an important mechanism causing continuous loss of myocytes and lead to myocardial dysfunction. The genetic testing of LMNA gene should be offered, because of the high risk of sudden death in these patients. It could be needed for new strategies to suppress and/or prevent inappropriate cardiac cell death in patients carrying LMNA mutation as a therapeutic means of slowing down the loss of myocytes.

## Materials and Methods

### Generation of the transgenic mice

The G→A substitution of LMNA cDNA (IMAGE: 2822703) that results in the E82K mutation in the protein was induced using the QuikChange site-directed mutagenesis kit (Stratagene, USA) and the sequence was confirmed by DNA sequencing. The mutated cDNA was cloned into an expression plasmid under the α-MHC promoter. The transgenic mice were generated by microinjection method [44]. Genotyping of transgenic mice was facilitated by the polymerase chain reaction (PCR) using the primers, 5′ AGAAGGAGGGTGACCTGATAG and 5′ ACCAGGTTGCTGTTCCTCT. The desired 490 bp fragment of the transgenic gene was amplified for 35 cycles at condition of 94°C for 30 s, 57°C for 30 s and 72°C for 30 s. The expression of the target gene was analyzed by western blot analysis using antibody to human LMNA (Santa Cruz). All the mice were bred in an AAALAC-accredited facility and the use of animals was approved by the Animal Care and Use Committees of The Institute of Laboratory Animal Science of Peking Union Medical College (GC08-2001).

### Light and electron microscopy

For light microscopy, cardiac tissue from mice at 6 months of age was fixed in formaldehyde and mounted in paraffin blocks. Sections were stained with Hematoxylin and Eosin (H&E) or Masson trichrome. For electron microscopy, cardiac tissue was routinely fixed in 2.5% glutaraldehyde in 0.1 M phosphate buffer (pH 7.4) and postfixed in 1% osmium tetroxide buffer for 1 hr. The sections were stained with uranyl acetate and lead citrate and examined under a JEM-1230 transmission electron microscope.

### Echocardiography

Mice were lightly anesthetized by intraperitoneal injection of tribromoethanol at a dose of 18 ml/kg body weight. M-mode echocardiography was performed at 2, 4, 6 and 8 months of age for each transgenic mouse with a 30 MHz transducer (Vevo770, Canada) [44], [45].

### Electrocardiography

Mice were fixed in the supine position on a heating pad to maintain core body temperature, and limb leads were place subcutaneously in accordance to chosen preferential derivation (lead II). Traces were recorded using a digital system (ADInstruments, USA) connected to a bioamplifier. The traces were analyzed using the LabChart software package (ADInstruments, USA) by an investigator who was blinded to the genotypes of the mice.

### Reverse transcription polymerase chain reaction (RT-PCR)

Total RNA was isolated from mice heart tissues using TRIzol Reagent (Invitrogen). First-strand cDNA was synthesized according to the Superscript III reverse transcriptase manufacturer' protocol (Invitrogen). The expression level of mRNA for ANP, BNP and Col3α1 was carried out by the RT-PCR and GAPDH was used as normalization (For ANP, 5′- ATGGGCTCCTTCTCCATCAC and 5′- TTATCTTCGGTACCGGAAGCTG; For BNP, 5′- ATGGATCTCCTGAAGGTGCTGTC and 5′- CTACAACAACTTCAGTGCGTTAC; for Col3α1, 5′- GGCAGTGATGGGCAACCT and 5′- TCCCTTCGCACCGTTCTT; for GAPDH, 5′- CAAGGTCATCCATGACAACTTTG and 5′- GTCCACCACCCTGTTGCTGTAG).

### Western blot

Total protein lysates from mice heart tissues were prepared by homogenizing with lysis buffer (50 mM Tris, pH 7.4, 150 mM NaCl, 1% Triton X-100, 1% sodium deoxycholate, 0.1% SDS, 1 mM EDTA, and protease inhibitor cocktail). After performing SDS-PAGE and transfer to nitrocellulose (Immobilon NC; Millipore), the membranes were incubated overnight with antibody to Lamin A/C (Santa Cruz); Actα1 (Abcam); FAS (Santa Cruz); caspase-3 (Cell Signaling); caspase-8 (Cell Signaling) or caspase-9 (Cell Signaling). After incubation with the appropriate secondary antibody for 1 h at room temperature, antibody binding was detected with a HRP-conjugated immunoglobulin G (Santa Cruz) using a chemiluminescent detection system (Westernblotting luminal reagent, Santa Cruz). GAPDH was served as normalization.

### Immunofluorescence

The sections of hearts were prepared in a standard pathological procedure. The sections were dewaxed, rehydrated, unmask the epitope, blocked, then incubated with anti-lamin A/C monoclonal Ab (Santa Cruz) overnight at 4°C Sections were washed with PBS and incubated with DyLight-conjugated, affinity-purified anti-mouse IgG (KPL) for 1 hr at room temperature, and all slides were counterstained with 300 nM 4,6-diamidino-2-phenylindole (DAPI, Invitrogen). After washing with PBS, sections were mounted in ProLong Gold antifade reagent (Invitrogen). Images of the sections were collected and analyzed under confocal laser scanning microscopy (Leica TCS SP2, Germany).

### TUNEL assay

TUNEL assay was performed in sections using an *In Site* Cell Death Detection Kit (Roche Diagnostics GmbH, Mannheim, Germany) principally according to the manufacturer's instructions. The sections of heart tissues were incubated with the TUNEL reaction mixture for 1 hr at 37°C in a dark, humidified chamber. Labeled DNA was visualized with an anti-fluorescein antibody conjugated with peroxidase (POD) using 3,3′-diaminobenzidine (DAB) as the chromogen. Sections were then washed, counterstained with hematoxylin. For negative control, TdT was omitted from the reaction mixture. Six images per heart (3 hearts per genotype group) were acquired, and positive cells were counted individually. Results were expressed as the percentage of apoptotic cells among the total cell population.

### Detection of mitochondrial cytochrome *c* release

A whole mouse heart from mice were excised and washed in cold PBS and the cytosolic and mitochondria fractions were derived following the Mitochondrial/Cytosol Fractionation Kit manufacturer's protocol (DBI Bioscience). The cytochrome *c* content in cytosol and mitochondria was detected by western blot analysis using antibody to cytochrome *c* (Cell Signaling). GAPDH was served as normalization.

### Statistical Analysis

Data was analyzed with unpaired two-tailed Student's t-tests for two groups, or one-way ANOVA for multiple groups followed by a Tukey's post hoc analysis. Data were expressed as mean ± SEM from individual experiments. Differences were considered as significant at *P*<0.05.

## Supporting Information

Table S1 **Echocardiographic characteristics of WT and** *Lmna* **^E82K^** **transgenic mice at 2, 4 and 8 months of age.** LV: left ventricular; LVEDD: LV end‐diastole diameter; LVESD: LV end‐systole diameter; LVEDV: LV end‐diastolic volume; LVESV: LV end‐systole volume; LVPWD: LV posterior wall at end‐diastole; LVPWS: LV posterior wall at end‐systole; LVAWD: LV anterior wall at end‐diastole; LVAWS: LV anterior wall at end‐systole; EF%: percent ejection fraction; FS%: percent fractional shortening; HR: heart rate. *P<0.05 *versus* NTG mice; ^‡^ *P*<0.01 *versus* NTG mice; ^#^ *P*<0.001 *versus* NTG mice.
(DOC)
