# Supplementary material for: LMNA E82K Mutation Activates FAS and Mitochondrial Pathways of Apoptosis in Heart Tissue Specific Transgenic Mice
Source: PLoS One. 2010 Dec 6;5(12):e15167. doi: 10.1371/journal.pone.0015167 (PMC2997782; doi:10.1371/journal.pone.0015167)
Supplement: Table S1 — Echocardiographic characteristics of WT and Lmna E82K transgenic mice at 2, 4 and 8 months of age. LV: left ventricular; LVEDD: LV end‐diastole diameter; LVESD: LV end‐systole diameter; LVEDV: LV end‐diastolic volume; LVESV: LV end‐systole volume; LVPWD: LV posterior wall at end‐diastole; LVPWS: LV posterior wall at end‐systole; LVAWD: LV anterior wall at end‐diastole; LVAWS: LV anterior wall at end‐systole; EF%: percent ejection fraction; FS%: percent fractional shortening; HR: heart rate. *P<0.05 versus NTG mice; ‡ P<0.01 versus NTG mice; # P<0.001 versus NTG mice. (DOC) [file pone.0015167.s001.doc]

**Table S1.** Echocardiographic characteristics of WT and *Lmna*^E82K^ transgenic mice aged 2, 4 and 8 months

| **Age** |  | **2 months** |  |  | **4 months** |  |  | **8 months** |  |
| --- | --- | --- | --- | --- | --- | --- | --- | --- | --- |
| **Mice** | **NTG** | ***Lmna*^E82K^ (F_0_30)** | ***Lmna*^E82K^ (F_0_35)** | **NTG** | ***Lmna*^E82K^ (F_0_30)** | ***Lmna*^E82K^ (F_0_35)** | **NTG** | ***Lmna*^E82K^ (F_0_30)** | ***Lmna*^E82K^ (F_0_35)** |
| **Number of mice** | 10 | 10 | 8 | 12 | 10 | 11 | 15 | 14 | 14 |
| **LVIDD, mm** | 3.63 ±0.26 | 3.98 ±0.25 ^‡^ | 4.01 ±0.15 ^‡^ | 3.78 ±0.22 | 4.23 ±0.13^#^ | 4.29±0.23^#^ | 4.02 ±0.27 | 4.69 ±0.39^#^ | 4.59 ±0.22*** |
| **LVIDS, mm** | 2.48±0.29 | 2.98±0.26^#^ | 2.99±0.19^#^ | 2.56±0.23 | 3.26±0.15^#^ | 3.29±0.21^#^ | 2.80±0.29 | 3.75±0.53^#^ | 3.77±0.29*** |
| **LVEDV, μL** | 56.12 ±9.72 | 69.41 ± 10.21 ^‡^ | 70.70 ±6.26 ^‡^ | 61.61 ±8.62 | 80.20 ± 5.74^#^ | 83.10 ±10.38^#^ | 71.32 ±11.28 | 103.07 ±20.06^#^ | 97.05±11.04*** |
| **LVESV, μL** | 22.48 ±6.14 | 35.02 ±7.41^#^ | 35.10 ±5.75^#^ | 23.96 ±5.36 | 43.16 ±4.95^#^ | 44.21 ±6.83^#^ | 30.12 ±7.62 | 61.85 ±19.17^#^ | 61.38 ±10.52*** |
| **LVPWD, mm** | 0.63 ±0.07 | 0.56 ±0.06* | 0.54 ±0.07* | 070 ±0.10 | 0.59±0.07 ^‡^ | 0.57±0.06^#^ | 0.67 ±0.08 | 0.54 ±0.10^#^ | 0.55 ±0.08*** |
| **LVPWS, mm** | 0.82 ±0.13 | 0.70 ±0.09* | 0.68 ±0.07* | 0.94 ±0.13 | 0.72 ±0.06^#^ | 0.71 ±0.06^#^ | 0.91 ±0.09 | 0.71 ±0.12^#^ | 0.67 ±0.09*** |
| **LVAWD, mm** | 0.74 ±0.11 | 0.68 ±0.06* | 0.64 ±0.07* | 0.80 ±0.10 | 0.66 ±0.02^#^ | 0.64 ±0.05^#^ | 0.73 ±0.10 | 0.61 ±0.08^#^ | 0.61 ±0.11 ^‡^ |
| **LVAWS, mm** | 0.93 ±0.11 | 0.80 ±0.10* | 0.80 ±0.04 ^‡^ | 1.01 ±0.07 | 0.78 ±0.05^#^ | 0.79 ±0.08^#^ | 0.95±0.13 | 0.78±0.22* | 0.70±0.12*** |
| **EF%** | 60.33 ±7.64 | 49.89 ±5.09 ^‡^ | 50.55 ±5.64 ^‡^ | 61.24 ±6.59 | 46.50 ±3.60^#^ | 46.90 ±4.09^#^ | 58.06 ±7.00 | 40.94 ±10.54^#^ | 36.89 ±6.90*** |
| **FS%** | 31.77 ±5.32 | 25.05±3.18 ^‡^ | 25.47 ±3.47* | 32.47 ±4.47 | 23.06±2.12^#^ | 23.36±2.45^#^ | 30.45 ±4.76 | 20.31 ±6.49^#^ | 17.81 ±3.95*** |
| **HR, bpm** | 435.25 ±42.70 | 410.10±48.15 | 411.88 ±51.72 | 403.75 ±44.08 | 453.00±39.95 | 421.82±65.91 | 484.47 ±63.47 | 415.58±50.89 | 411.29 ±84 |

LV: left ventricular; LVEDD: LV end-diastole diameter; LVESD: LV end-systole diameter; LVEDV: LV end-diastolic volume; LVESV: LV end-systole volume; LVPWD: LV posterior wall at end-diastole; LVPWS: LV posterior wall at end-systole; LVAWD: LV anterior wall at end-diastole; LVAWS: LV anterior wall at end-systole; EF%: percent ejection fraction; FS%: percent fractional shortening; HR: heart rate. **P* <0.05 *versus* NTG mice; ^‡^ *P* <0.01 *versus* NTG mice; ^#^*P* <0.001 *versus* NTG mice.
